# Successful resection of giant esophageal liposarcoma by endoscopic submucosal dissection combined with surgical retrieval: a case report and literature review

**DOI:** 10.1186/s40792-016-0219-5

**Published:** 2016-09-02

**Authors:** Gosuke Takiguchi, Tetsu Nakamura, Yasunori Otowa, Ayako Tomono, Shingo Kanaji, Taro Oshikiri, Satoshi Suzuki, Tsukasa Ishida, Yoshihiro Kakeji

**Affiliations:** 1 Division of Gastrointestinal Surgery, Department of Surgery, Kobe University Graduate School of Medicine, 7-5-2 Kusunoki-chou, Chuo-ku, Kobe, Hyogo 650-0017 Japan; 2 Division of Gastroenterology, Department of Internal Medicine, Kobe University Graduate School of Medicine, Kobe, Hyogo 650-0017 Japan

**Keywords:** Esophagus, Liposarcoma, ESD, Esophagotomy, Combined method

## Abstract

Liposarcoma of the esophagus is very rare. We experienced a huge (27.5 × 11.6 cm) liposarcoma of the esophagus. A 73-year-old man presented with severe dyspnea requiring emergency tracheal intubation. Computed tomography and esophagogastroduodenoscopy showed a large submucosal tumor arising from the esophageal entrance and extending intraluminally to the lower esophagus. We successfully performed endoscopic submucosal dissection (ESD) and esophagotomy to remove the tumor, which preserved swallowing and phonation. The final diagnosis by histopathologic and immunohistologic examination was well-differentiated liposarcoma of the esophagus. Treatment by the combination of ESD and esophagotomy can be performed even for a very large tumor. This method preserves deglutition with a lower risk of recurrent laryngeal nerve paralysis than that with esophagectomy.

## Background

Liposarcoma of the gastrointestinal tract is rare, with a reported incidence at autopsy of 0.1 to 5.8 %. It is particularly rare in the esophagus, accounting for 1.2 to 1.5 % of all gastrointestinal liposarcomas [1]. This tumor is most commonly a pedunculated polyp and is diagnosed as a submucosal tumor [2].

The most common presenting symptom of esophageal submucosal tumors is dysphagia. However, we saw a patient with esophageal liposarcoma who presented with severe dyspnea. Previously reported liposarcomas of the esophagus had been treated with various surgical methods, including simple resection by the transcervical, transthoracic, or transabdominal route. However, such approaches are invasive and expensive. The first report of endoscopic resection of the esophageal liposarcoma was reported in 2007, with endoscopic submucosal dissection (ESD) first reported in 2013 [3, 4].

We report a huge liposarcoma of the esophagus that compressed the airway and was successfully resected by combined ESD and esophagotomy.

## Case presentation

A 73-year-old man was brought to the emergency room with severe dyspnea. His height was 170 cm and weight 78 kg. He had a history of hypertension and atrial fibrillation and had undergone surgery for a subarachnoid hemorrhage 20 years earlier.

**Fig. 1:**
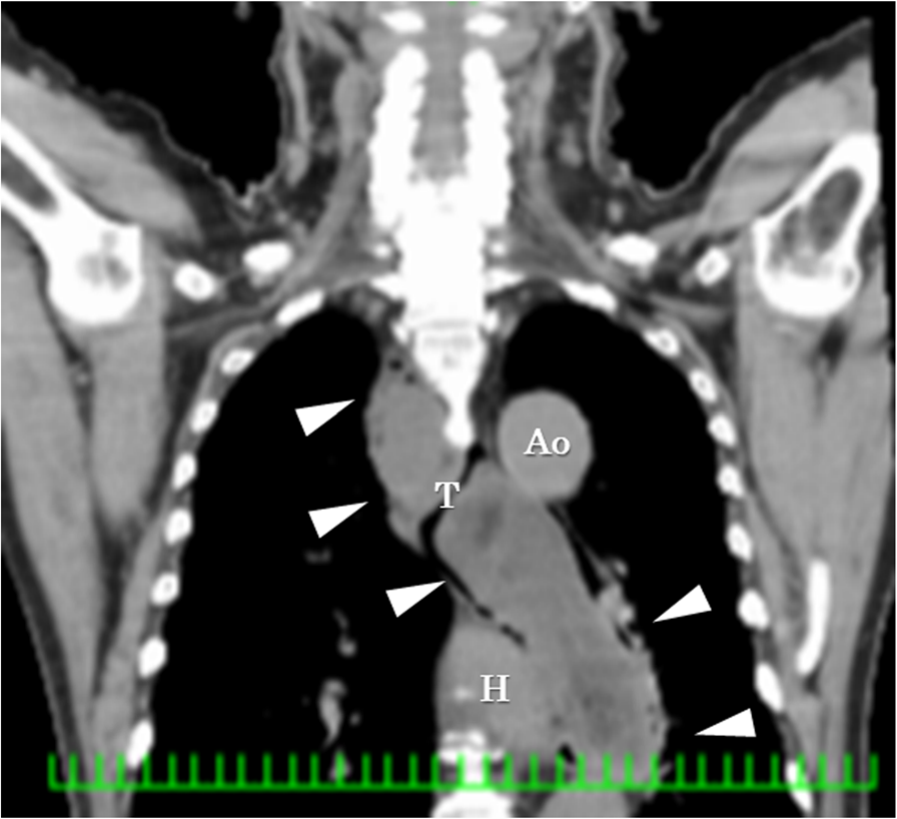
Computed tomography showed giant tumor from the pyriform sinus into the thoracic esophagus intraluminal of the esophagus. The tumor density was mostly low with heterogeneous density in spots (*arrow head*, *T* tumor, *H* heart, *Ao* aorta)

Several hours before presentation, he had temporary difficulty in swallowing which resolved after drinking some water. However, he suddenly had difficulty breathing and called emergency medical services. On arrival at the emergency room, he was given oxygen and a bronchodilator, which failed to improve the dyspnea. Computed tomography (CT) showed a giant intraluminal tumor from the pyriform sinus into the thoracic esophagus (Fig. 1). The tumor density was mostly low, but there was heterogeneous density in spots. No metastatic lesion was detected by CT. The membranous portion of the trachea was compressed by the tumor, narrowing the airway and causing stridor. The oxygenation level was dropping, so emergency tracheal intubation was performed.

**Fig. 2:**
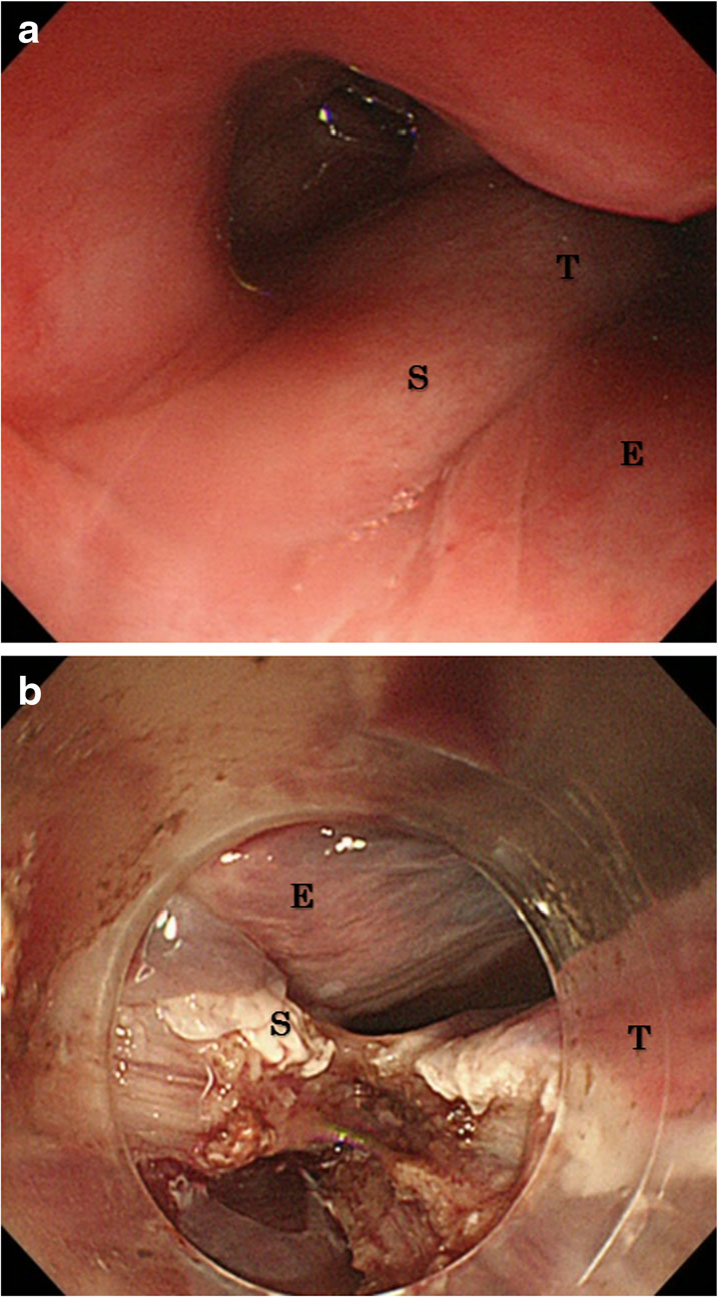
**a** Esophagogastroduodenoscopy showed that the stalk of the tumor was from the esophageal entrance to oropharynx and the surface was covered with smooth mucous membrane. **b** The stalk was resected from the base in the endoscopic submucosal dissection procedure (*T* tumor, *S* stalk, *E* esophagus)

**Fig. 3:**
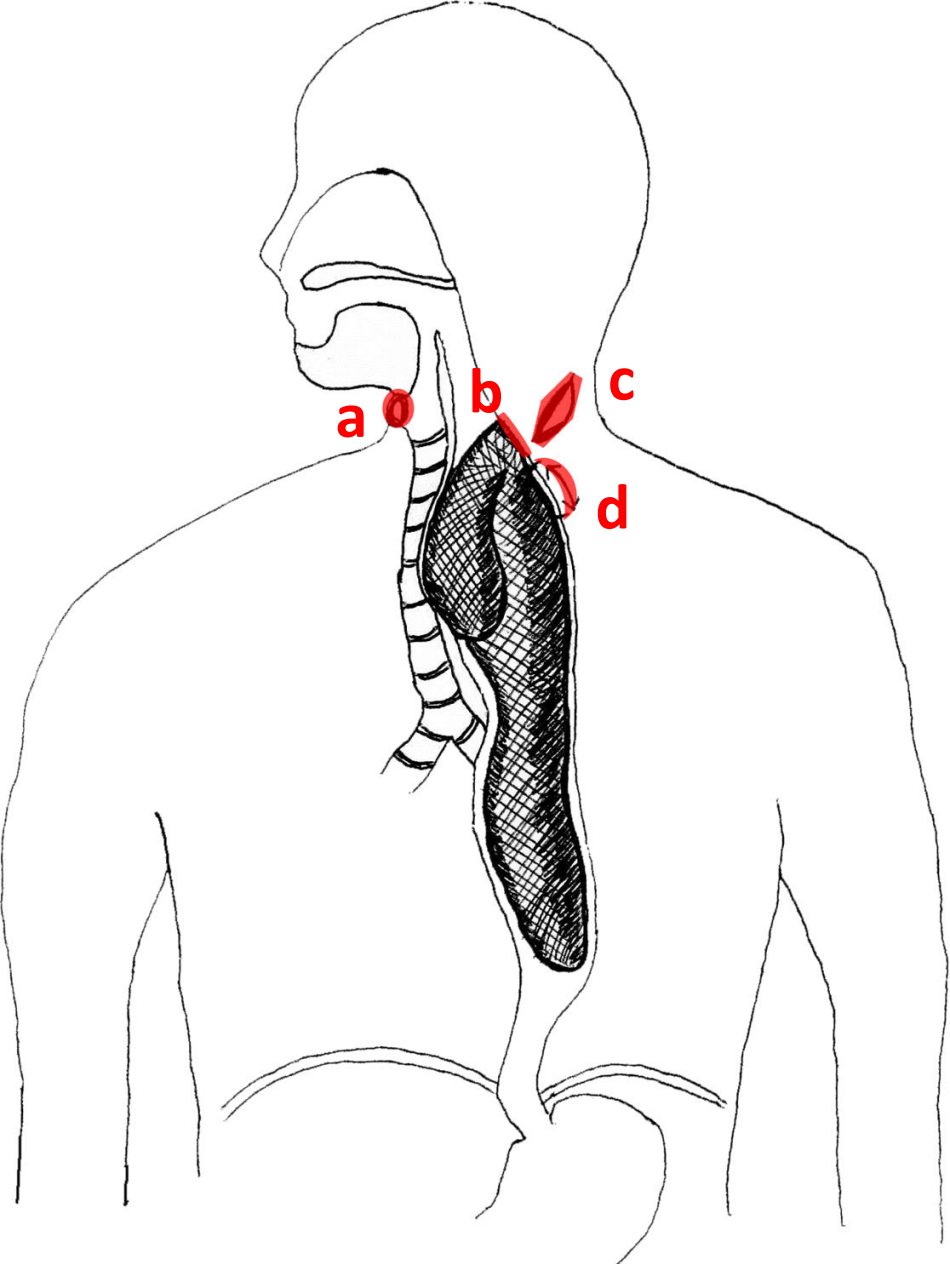
**a** Tracheotomy was done for keeping the airway, which was compressed by the tumor. **b** The stalk of the tumor was easily observed at the cervical esophagus. Endoscopic submucosal dissection was performed, and the tumor was excised from the base. **c** Skin incision was made at the left neck because the tumor was too large to retrieve from the mouth. **d** Esophagotomy was performed at cervical region for about 5 cm, and the tumor was retrieved from this incised part

Esophagogastroduodenoscopy (EGD) showed the stalk of the tumor at the esophageal entrance, having a surface covered with a smooth mucous membrane (Fig. 2a). These findings strongly suggested a submucosal soft tissue tumor of the esophagus, particularly liposarcoma. After tracheotomy was successfully performed, the patient was transferred to our hospital for further treatments (Fig. 3a).

Our treatment strategy for this huge esophageal tumor was designed to achieve curative resection while preserving swallowing and phonation. It appeared that a minimally invasive method without resecting the esophagus was possible because the tumor had a stalk that was confirmed by EGD, that is, it was a pedunculated submucosal tumor.

ESD was selected as main treatment (Figs.2b and 3b). ESD was performed with a Flush Knife-BT (DK-2618JN; FTS, Tokyo, Japan), through a single-channel endoscope with water-jet function (GIFQ260J; Olympus, Tokyo, Japan). The length of the Flush Knife-BT was 2.0 mm, and ICC 200 (ERBE, Elektromedizin, Tubingen, Germany) was used as the power source for electrical cutting and coagulation. None of the findings on the diagnostic workup had suggested invasion below the submucosa, and it was in fact easy to exfoliate the submucosal layer.

**Fig. 4:**
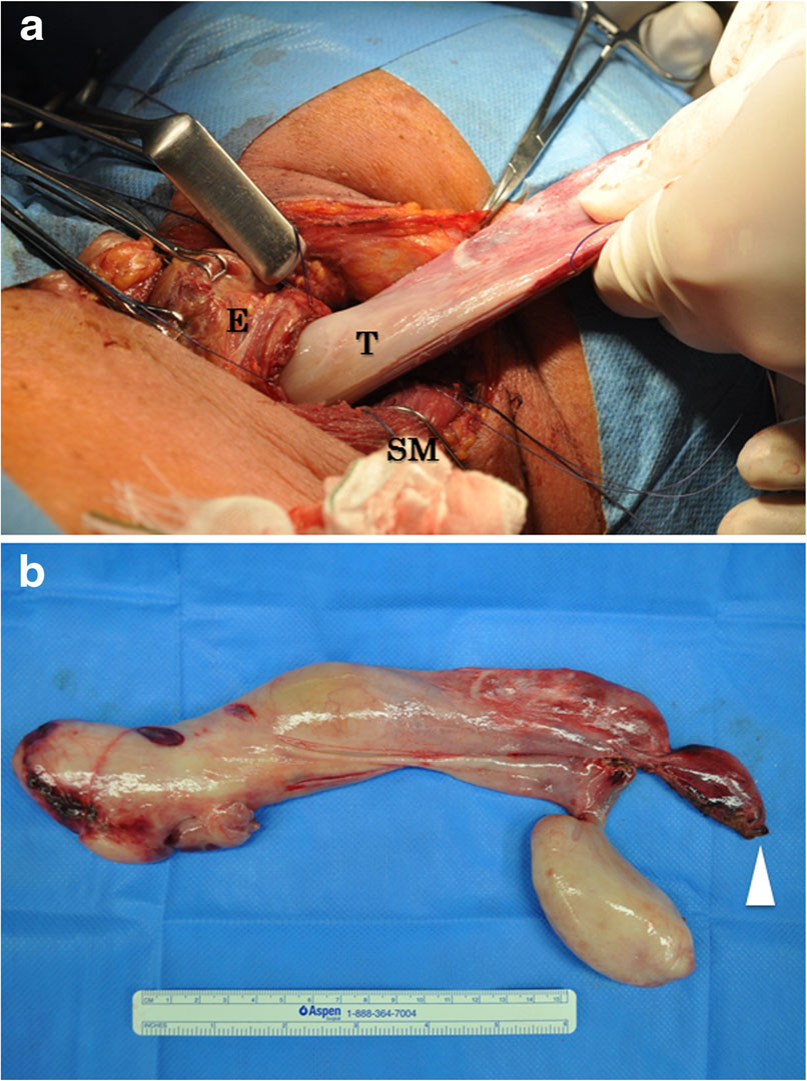
Excised specimen. **a** The skin incision was made at the left neck. Esophagotomy at cervical region for about 5 cm was performed to retrieve the tumor. **b** The excised specimen consisted of 27 × 11.6 cm branch polypoid mass. Surface of this tumor was smooth and white-colored (*arrow head* edge of the stalk, *T* tumor, *E* esophagus, *SM* sternocleidomastoid muscle)

Since the tumor was too large to retrieve through the mouth, we made an oblique skin incision at the left neck (Fig. 3c), followed by a 5-cm esophagotomy, through which the entire tumor was removed (Figs. 3d and 4a).

The postoperative course was uneventful. The patient had no difficulty swallowing and tolerated oral intake well. There were no signs suggestive of recurrent laryngeal nerve paralysis. After 41 months of postoperative follow-up, the patient remained in good condition with no evidence of recurrence.

**Fig. 5:**
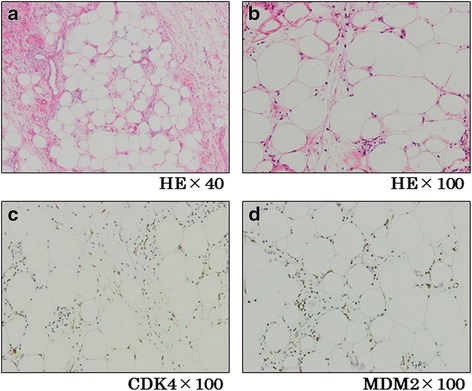
**a**, **b** Histopathological examination showed internal of the tumor consisted of adipose tissue, enriched small vessel, and lymph duct. **c**, **d** Immunohistologically, adipose cells were positive for CDK4 (**c**) and weakly positive for MDM2 (**d**) in nuclei (*HE* hematoxylin and eosin stain, *CDK4* cyclin-dependent kinase 4, *MDM2* murine double minute-2)

The excised specimen consisted of a 27.5 × 11.6-cm branched polypoid mass (Fig. 4b) with a smooth, white surface. The cut surface was yellow-white and edematous. Histopathologic examination (Fig. 5a, b) showed that the tumor was composed of adipose tissue, numerous small vessels, and lymphatics. The surface was covered by the normal squamous epithelium. Immunohistologically, these cells were positive for murine double minute-2 and weakly positive for cyclin-dependent kinase 4 in the nuclei (Fig. 5c, d); they were diffusely positive for p16. The final pathology diagnosis was well-differentiated liposarcoma of the esophagus. The surgical margins of the tumor were microscopically negative. No postoperative adjuvant therapy was given.

### Discussion

Liposarcoma is the most common soft tissue malignant tumor in adults and usually occurs in the retroperitoneum, deep soft tissues of the trunk, or lower extremities. It is very rare in the gastrointestinal tract, especially in the esophagus [5]. In PubMed search using the words “liposarcoma” and “esophagus,” we found only 33 reports from 1983 to 2015. Including two cases from our facility, we reviewed 35 cases of esophageal liposarcoma. The average age was 58.4 years, and the male-to-female ratio was approximately 3:1. The average size was 13.3 cm (range, 4–27 cm), and the most common symptom was dysphagia (88.6 %) (Table 1).

**Table 1:**
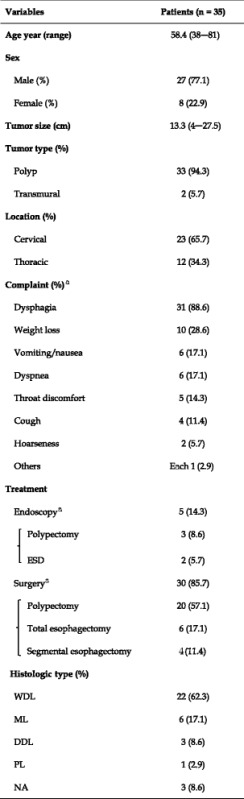
Characteristics of patients with esophageal liposarcoma

*WDL* well-differentiated liposarcoma, *DDL* dedifferentiated liposarcoma, *ML* myxoid liposarcoma, *PL* pleomorphic liposarcoma, *NA* not available

^a^Some patients had several symptoms

Our patient, conversely, complained of severe dyspnea resulting from compression of the membranous portion of the trachea by the huge tumor. Therefore, tracheotomy was required for airway management. None of the patients with esophageal liposarcoma reported in the literature had similar airway compression. There are, however, reports of sudden asphyxia due to pedunculated polyps of the esophagus or pharynx [6].

Since tumor resection is the only curative treatment for liposarcoma [7, 8], such lesions in the esophagus are usually surgically or endoscopically removed. Of the 35 cases we reviewed, surgical resections, either polypectomy or esophagectomy, were performed in 30 patients (85.7 %), endoscopic polypectomy in three (8.6 %), and ESD in two (5.7 %). The average tumor size was 15.2 cm for cases resected by total esophagectomy and 6.7 cm for those removed by endoscopic polypectomy; so the larger the tumor, the more likely the need for surgery.

Liposarcoma originates from a primitive mesenchymal cell and usually arises from the esophageal mucosa and submucosa [9, 10]. According to the literature review, 94 % or reported tumor was polypoid, and 68 % arose from the cervical esophagus (Table 1). When the base of the tumor is clearly demonstrated endoscopically, ESD enables curative resection by removing the tumor base. Intraoperative pathology examination may help to confirm negative surgical margins. In our patient, ESD successfully removed the tumor, but the entire lesion was too large to be removed through the mouth. Performing esophagotomy through a neck incision after ESD allowed the tumor to be removed in its entirely without subsequent compromise of swallowing or phonation.

Generally, the aggressiveness of soft tissue sarcoma differs depending on histologic type. Enzinger reported that the patients with well-differentiated liposarcoma in various organs had the highest 5-year survival and the lowest local recurrence rate [11]. But the prognosis after resection of an esophageal liposarcoma is unclear. To the best our knowledge, there have been only two reported cases of recurrence at 78 and 300 months after surgery [12, 13]. The tumors in these cases were well-differentiated, myxoid lesions. In those two cases, there was no information on the surgical margins. In general, esophageal liposarcoma is a slow-growing tumor [12], so a long period of follow-up may be advisable to detect late recurrence.

## Conclusions

We successfully treated a large esophageal liposarcoma by combining ESD and esophagotomy. We think this combined method is safety and less invasive than esophagectomy, but only as long as the base of the stalk is confirmed by EGD to be in the cervical esophagus. This method can theoretically be performed regardless of the tumor size. Moreover, this method enables preservation of deglutition so that the patient can resume oral intake. Such a combined procedure also carries a lower risk of recurrent laryngeal nerve paralysis than dose esophagectomy.

## Abbreviations

CT: Computed tomography
EGD: Esophagogastroduodenoscopy
ESD: Endoscopic submucosal dissection
